# State Registration

**Published:** 1856-05

**Authors:** 


*State Registration.*—An order was adopted in the State Senate, on Friday last, directing the Secretary of the Commonwealth, at the close of the session, to prepare and distribute to the clerks and registers of cities and towns, clergymen, &c., a circular, setting forth the duties of registers of births, deaths and marriages, with the penalties for not conforming to the requirements of the law. We hope this will have the effect of producing greater accuracy in the returns, and consequently of adding to the value of the Massachusetts Registration Reports.—*Ibid.*

